# Staged traditional Chinese medicine treatment for suspected pulmonary inflammatory pseudotumor with progressive lesion reduction: a case report and literature review

**DOI:** 10.3389/fmed.2026.1873013

**Published:** 2026-08-20

**Authors:** Saimei Nie, Bei Wang, Chunyan Su

**Affiliations:** Department of Critical Care Medicine, Wangjing Hospital of China Academy of Chinese Medical Sciences, Beijing, China

**Keywords:** case report, conservative management, pulmonary inflammatory pseudotumor, syndrome differentiation, traditional Chinese medicine

## Abstract

**Background:**

Pulmonary inflammatory pseudotumor (PIPT) is a rare benign lesion often indistinguishable from malignancy on imaging. For indeterminate solid masses exceeding 3 cm, surgical resection is generally recommended. Conservative management strategies for patients who decline surgery remain poorly defined.

**Case presentation:**

A 55-year-old male presented with a 3.4 cm × 2.6 cm irregular mass in the right lower lobe with lobulation and small bubble signs. CT-guided percutaneous biopsy revealed fibrous tissue proliferation with lymphoplasmacytic infiltration and epithelioid granulomas without necrosis. Immunohistochemistry showed TTF-1 (+), NapsinA (+), P40 (focally +), P53 (wild-type pattern), CEA (−), Ki67 (∼10%), KP-1 (+), CD38 (+), IgG (+), and IgG4 (focally +); special stains were negative. An inflammatory process consistent with PIPT was suspected; however, ALK, SMA, and Vimentin were not tested, and definitive distinction from pulmonary inflammatory myofibroblastic tumor (PIMT) could not be established. The patient declined surgery and received staged traditional Chinese medicine (TCM) treatment: early-phase therapy focused on dispersing wind and ventilating the lung; subsequent phases transitioned to supplementing Qi (vital energy), resolving phlegm, and activating blood circulation. Serial CT (same institution/scanner) demonstrated progressive lesion reduction to 2.5 cm × 1.8 cm at 5 months and 1.9 cm × 0.7 cm at 10 months, with corresponding symptomatic improvement.

**Conclusion:**

Spontaneous regression cannot be excluded. However, the absence of corticosteroid use, the pre-treatment stability of the lesion, and the stepwise reduction observed during successive treatment phases are temporally consistent with a treatment-associated response. Staged TCM syndrome differentiation may warrant further investigation as a conservative option for selected patients with inflammatory pulmonary lesions who decline surgery.

## Introduction

Pulmonary space-occupying lesions have complex pathological characteristics and encompass a broad spectrum of pathological conditions, including benign and malignant tumors as well as inflammatory diseases (1). Among these benign lesions, pulmonary inflammatory pseudotumor (PIPT) is a commonly used descriptive pathological term in clinical practice. PIPT must be distinguished from pulmonary inflammatory myofibroblastic tumor (PIMT), which is classified as a distinct pathological entity with intermediate biological behavior under the 2021 WHO classification and carries a recognized risk of local recurrence and occasional metastatic potential (2, 3). The imaging features of PIPT often include lobulation, spiculation, or pleural traction, making it challenging to distinguish from lung cancer, tuberculoma, and other pulmonary space-occupying lesions (4–6). For solid pulmonary masses larger than 3 cm with an indeterminate nature, diagnostic surgical resection is recommended in current clinical consensus to reduce the risk of missed diagnosis when biopsy findings are inconsistent with imaging features highly suggestive of malignancy (1, 7, 8). However, for patients who refuse invasive treatment, optimal conservative management strategies remain poorly defined. We present this case in accordance with the CARE Guidelines. The intervention followed a structured, three-phase protocol in which syndrome differentiation was formally reassessed and the herbal formula adjusted at each follow-up visit; CT imaging performed at the same intervals served as an objective measure of lesion change. This report aims to provide a clinical reference for conservative PIPT management when surgery is declined and to inform future prospective studies.

## Case presentation

**FIGURE 1:**
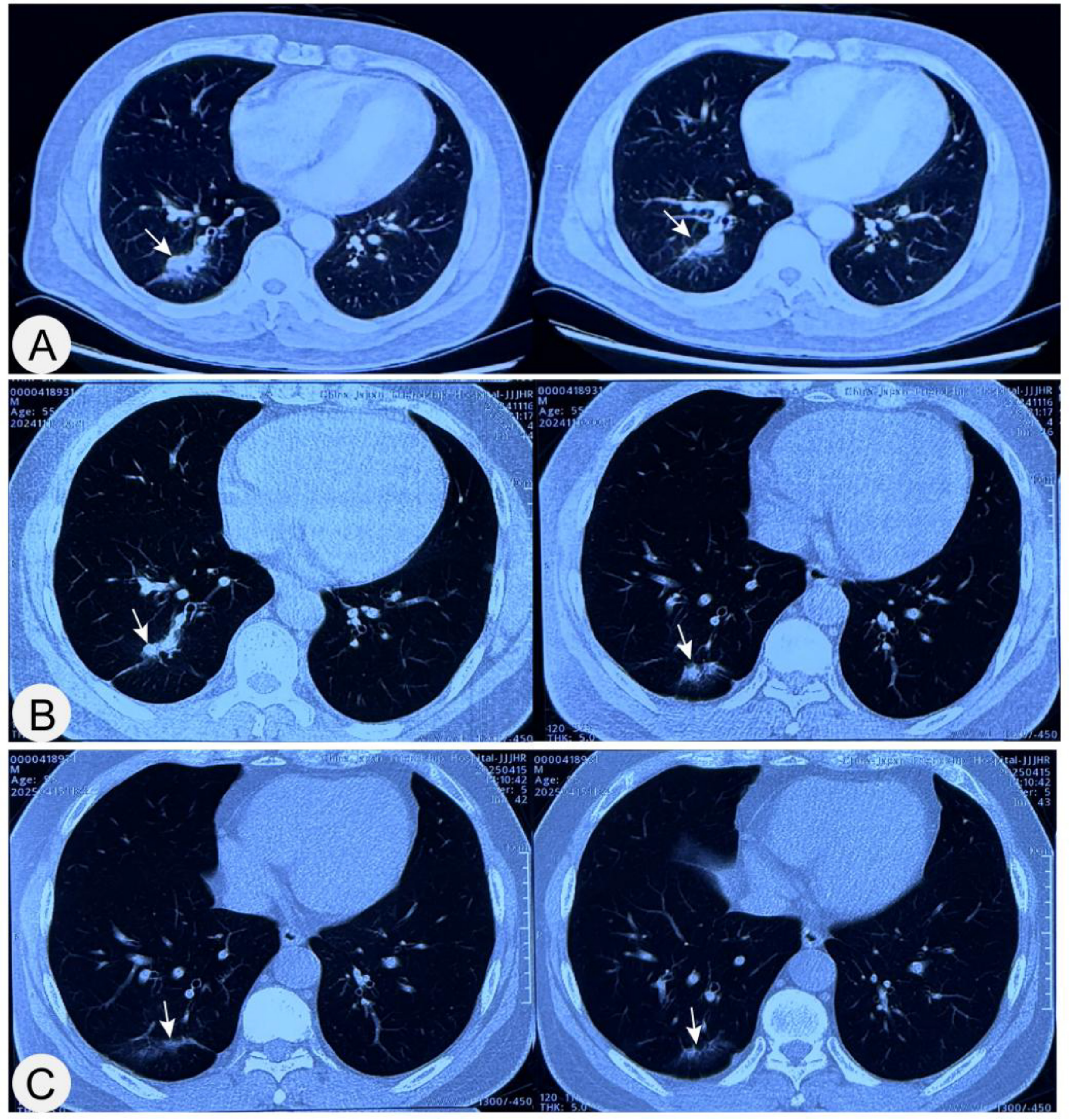
Comparison of chest CT images for the pulmonary lesion before and after treatment. **(A)** Baseline chest CT before treatment (June 14, 2024); **(B)** Chest CT after 4 months of treatment (November 16, 2024); **(C)** Chest CT after 9 months of treatment (April 15, 2025).

A 55-year-old male developed intermittent dry cough following an upper respiratory tract infection in April 2024. Chest computed tomography (CT) performed at China-Japan Friendship Hospital on April 25, 2024 revealed a mass in the right lower lobe measuring approximately 3.4 cm × 2.6 cm with multiple calcified foci, and tuberculoma was initially considered. Bilateral hila were not enlarged, no mediastinal lymphadenopathy was identified, and the pleural surfaces were unremarkable. These findings remained consistent across all subsequent CT examinations. In June 2024, the patient was hospitalized at the same institution, where contrast-enhanced chest CT and CT-guided percutaneous lung biopsy were performed. CT demonstrated an irregular nodule in the right lower lobe measuring 3.4 cm × 2.6 cm with lobulation and small bubble signs, showing low enhancement on contrast-enhanced imaging (Figure 1A). Histopathological examination revealed loss of normal alveolar architecture, fibrous tissue proliferation with marked lymphocytic and plasma cell infiltration, focal alveolar epithelial hyperplasia, and epithelioid granulomas without definite necrosis. Immunohistochemical staining showed TTF-1 (+), NapsinA (+), P40 (focally +), P53 (wild-type pattern), CEA (−), Ki67 (approximately 10%), KP-1 (+), CD38 (+), IgG (+), and IgG4 (focally +). Special stains including periodic acid-Schiff (PAS), acid-fast, and silver stains were all negative. TTF-1 and NapsinA positivity was interpreted as reflecting reactive type II pneumocyte hyperplasia rather than neoplastic proliferation, consistent with the histological finding of focal alveolar epithelial hyperplasia. Based on these findings, malignancy and tuberculosis were not supported. Rheumatologic and immunologic workup, including serum IgG subclass quantification, revealed no significant abnormalities. An inflammatory pulmonary space-occupying lesion was considered, and PIPT was clinicopathologically suspected. Surgical resection was recommended to establish a definitive diagnosis; however, the patient declined and subsequently presented to the Department of General Internal Medicine at our hospital seeking conservative treatment with TCM on July 20, 2024.

The patient had a 6-year history of hypertension managed with irbesartan/hydrochlorothiazide (one tablet daily). He denied any history of smoking or alcohol consumption, and no other chronic diseases were reported. At presentation, the patient had intermittent dry cough aggravated at night and by cold exposure, occasionally accompanied by white sputum. He also reported aversion to wind and sensitivity to cold. Appetite was normal, sleep was disturbed, and intermittent hemorrhoidal bleeding was noted. Bowel and urinary functions were normal. Physical examination revealed no abnormal findings. On TCM examination, the tongue was enlarged with teeth marks, pale red with a white coating, and the pulse was deep. The TCM diagnosis was cough due to wind pathogen invading the lung.

The treatment principle was dispersing wind, ventilating the lung, resolving phlegm, and relieving cough. A modified Zhisou Powder (止嗽散), a classical formula for prolonged cough following external pathogen invasion, was prescribed. The formula was composed of herbs for restoring lung dispersing and descending functions, dispersing wind and relieving the exterior, resolving phlegm and regulating Qi, supplemented by herbs for clearing heat and softening masses, with Huangqi (*Astragali Radix*) for supplementing Qi (the complete formula composition and dosages are presented in Table 1). The herbs were administered as formula granules (manufactured by Sichuan New Green Pharmaceutical Technology Development Co., Ltd., Sichuan, China) dispensed by the hospital granule pharmacy according to each prescription, one dose daily dissolved in 200 mL warm water, taken in two divided doses for 14 days.

**TABLE 1:** Herbal formula compositions across three treatment phases.

Chinese name	Pharmaceutical name	Phase I (Jul 2024)	Phase II (Nov 2024)	Phase III (Apr 2025)
Restoring lung dispersing and descending functions				
Jiegeng	*Platycodonis Radix*	15 g	15 g	15 g
Baiqian	*Cynanchi Stauntonii Rhizoma et Radix*	15 g	–	–
Ziwan	*Asteris Radix et Rhizoma*	15 g	–	–
Baibu	*Stemonae Radix*	10 g	–	–
Kuxingren	*Armeniacae Semen Amarum*	10 g	–	–
Dispersing wind and relieving the exterior				
Jingjie	*Schizonepetae Herba*	10 g	–	–
Bohe	*Menthae Haplocalycis Herba*	6 g	–	–
Chantui	*Cicadae Periostracum*	12 g	–	–
Resolving phlegm and regulating Qi				
Chenpi	*Citri Reticulatae Pericarpium*	10 g	12 g	12 g
Fuling	*Poria*	15 g	15 g	30 g
Lugen	*Phragmitis Rhizoma*	15 g	–	–
Wuweizi (vinegar-processed)	*Schisandrae Chinensis Fructus*	15 g	–	–
Fabanxia	*Pinelliae Rhizoma Praeparatum*	–	12 g	12 g
Gualou	*Trichosanthis Fructus*	–	15 g	15 g
Chaozhishi	*Aurantii Fructus Immaturus Praeparatus*	–	15 g	15 g
Zhebeimu	*Fritillariae Thunbergii Bulbus*	–	–	15 g
Clearing heat and dissipating masses				
Zaojiaoci	*Gleditsiae Spina*	15 g	15 g	–
Baijiangcao	*Patriniae Herba*	15 g	–	–
Xiakucao	*Prunellae Spica*	–	18 g	18 g
Activating blood and unblocking collaterals				
Danggui	*Angelicae Sinensis Radix*	–	12 g	15 g
Chuanxiong	*Chuanxiong Rhizoma*	–	9 g	9 g
Sigualuo	*Luffae Retinervus*	–	12 g	12 g
Puhuang	*Typhae Pollen*	–	12 g	12 g
Supplementing Qi and fortifying the spleen				
Huangqi	*Astragali Radix*	15 g	24 g	24 g
Baihe	*Lilii Bulbus*	–	30 g	30 g
Fuchaoyiyiren	*Coicis Semen Praeparatum*	–	–	30 g
Nourishing the heart and calming the mind				
Chaosuanzaoren	*Ziziphi Spinosae Semen Praeparatum*	–	–	45 g
Regulating Qi and soothing the liver				
Yujin	*Curcumae Radix*	–	–	15 g
Harmonizing the formula				
Gancao	*Glycyrrhizae Radix*	6 g	–	–
Zhigancao	*Glycyrrhizae Radix Praeparata*	–	9 g	9 g

Phase I: Modified Zhisou Powder for dispersing wind, ventilating the lung, resolving phlegm, and relieving cough. Phase II: Revised formula for resolving phlegm, reducing masses, and promoting blood circulation to dissipate nodules. Phase III: Further modified formula for resolving phlegm, eliminating blood stasis, and reinforcing healthy Qi to consolidate therapeutic effect. – indicates the herb was not included in this phase. All herbs were administered as formula granules (Sichuan New Green Pharmaceutical Technology Development Co., Ltd., Sichuan, China) dispensed by the hospital granule pharmacy, one dose daily dissolved in 200 mL warm water, taken in two divided doses for 14 days per treatment cycle.

From August to October 2024, cough and expectoration were markedly relieved, and hemorrhoidal bleeding ceased. Based on syndrome differentiation, Xiakucao (*Prunellae Spica*) and Fabanxia (*Pinelliae Rhizoma Praeparatum*) were added to the original prescription to strengthen the effects of resolving phlegm and dissipating masses. On November 19, 2024, the patient reported further improvement in cough with no obvious expectoration. The tongue remained enlarged with teeth marks, sublingual veins were distended, and the pulse was deep. Chest CT re-examination at China-Japan Friendship Hospital (November 16, 2024) revealed an irregular nodule in the right lower lobe measuring 2.5 cm × 1.8 cm, with lobulation and spiculation (Figure 1B), decreased in size compared with the previous imaging on June 14, 2024 (3.4 cm × 2.6 cm). The TCM diagnosis was updated to lung accumulation with phlegm and blood stasis interconnection. The treatment principle was adjusted to resolving phlegm, reducing masses, and promoting blood circulation to dissipate nodules. The revised prescription centered on herbs for resolving phlegm and dissipating masses, herbs for activating blood and unblocking collaterals, and herbs for supplementing Qi and fortifying the spleen (complete formula composition and dosages are presented in Table 1). Fourteen doses were prescribed with the same method of administration.

**FIGURE 2:**
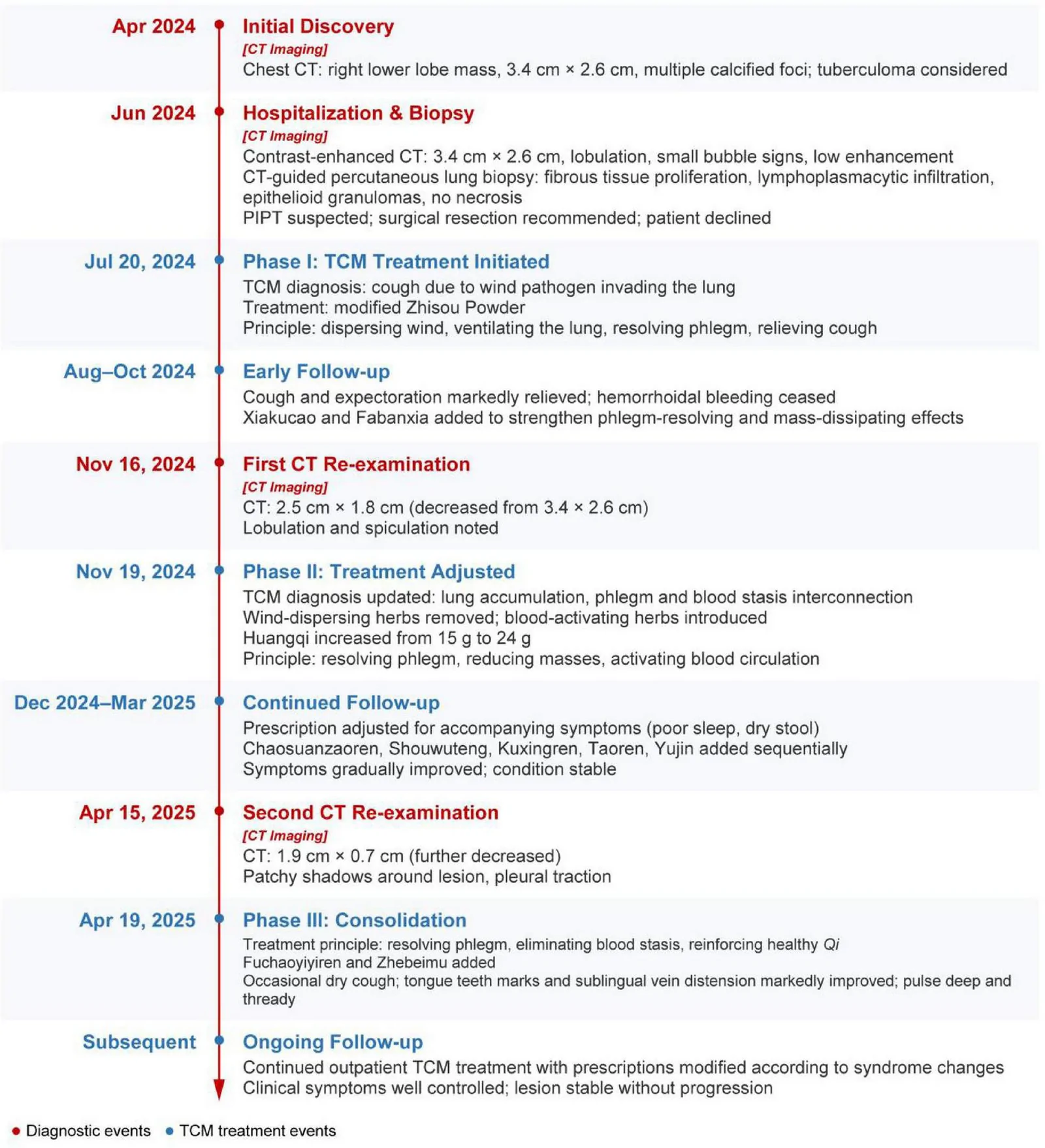
Clinical timeline of diagnosis, treatment, and follow-up. Lesion size progression: 3.4 cm × 2.6 cm (Jun 2024) → 2.5 cm × 1.8 cm (Nov 2024) → 1.9 cm × 0.7 cm (Apr 2025). All CT examinations were performed at China-Japan Friendship Hospital using the same scanner.

From December 2024 to March 2025, the prescription was further adjusted according to changes in symptoms. For accompanying manifestations including poor sleep and dry stool, Chaosuanzaoren (*Ziziphi Spinosae Semen Praeparatum*), Shouwuteng (*Polygoni Multiflori Caulis*), Kuxingren (*Armeniacae Semen Amarum*), Taoren (*Persicae Semen*), and Yujin (*Curcumae Radix*) were added in sequence to nourish the heart and calm the mind, moisten the intestines, regulate Qi, and promote blood circulation. The patient’s symptoms gradually improved and the condition remained stable. On April 19, 2025, the patient reported occasional dry cough. Sleep had improved, and bowel and urination were normal. The tongue remained enlarged, while teeth marks on the tongue margin and sublingual vein distension were markedly improved compared with previous visits. The pulse was deep and thready. Chest CT re-examination at China-Japan Friendship Hospital (April 15, 2025) showed that the irregular nodule in the right lower lobe had decreased to 1.9 cm × 0.7 cm, with patchy shadows around the lesion and pleural traction (Figure 1C), further reduced compared with imaging on June 14, 2024 (3.4 cm × 2.6 cm) and November 16, 2024 (2.5 cm × 1.8 cm). The treatment principle continued to focus on resolving phlegm, eliminating blood stasis, and reinforcing healthy Qi to consolidate the therapeutic effect (complete formula composition and dosages are presented in Table 1). Fourteen doses were prescribed with the same method of administration. The patient has continued outpatient TCM treatment, with clinical symptoms well controlled and the lesion stable on recent imaging. Figure 2 summarizes the clinical timeline from initial diagnosis to imaging follow-up.

## Discussion and literature review

The immunohistochemical profile did not support malignancy. TTF-1 and NapsinA are well-established markers of pulmonary adenocarcinoma, but both can also stain reactive type II pneumocytes, a known source of misinterpretation in non-neoplastic pulmonary lesions (9). In this case, their positivity was consistent with the concurrent histological finding of focal alveolar epithelial hyperplasia and was interpreted as reactive rather than neoplastic. Wild-type P53 expression, negative CEA staining, and a Ki67 proliferation index of approximately 10% further argued against a neoplastic process. KP-1 (CD68) and CD38 positivity indicated histiocytic infiltration and plasma cell involvement, consistent with chronic inflammation. P40 was only focally positive, providing no evidence for squamous cell carcinoma. PAS, Ziehl-Neelsen acid-fast, and Grocott’s methenamine silver (GMS) stains were all negative. T-SPOT.TB and tuberculin skin test results were likewise negative. Epithelioid granulomas were identified without definite necrosis. The negative special stain results (PAS, acid-fast, and silver stains) argued against granulomatous infection (10, 11). The non-necrotizing granulomatous pattern is, however, also consistent with sarcoidosis. Serial CT examinations demonstrated no bilateral hilar lymphadenopathy or mediastinal lymphadenopathy, and no multisystem involvement was observed, reducing the likelihood of systemic sarcoidosis. A localized sarcoid-like reaction could not be excluded without excisional biopsy and serum angiotensin-converting enzyme measurement. IgG4-related lung disease (IgG4-RLD) was subsequently evaluated and excluded. Immunohistochemistry revealed only scattered IgG4-positive plasma cells, falling below the diagnostic threshold of more than 10 positive cells per high-power field (HPF) and an IgG4/IgG ratio exceeding 40% (12). Serum IgG subclass quantification showed no elevation of the IgG4 fraction, and rheumatology consultation considered this diagnosis unlikely on the basis of the overall clinicopathological profile (13).

Distinguishing PIPT from pulmonary inflammatory myofibroblastic tumor (PIMT) required further consideration. The initial immunohistochemical panel did not include ALK, smooth muscle actin (SMA), or Vimentin, markers central to this distinction. ALK gene rearrangements are identified in approximately 50% of PIMT cases, and myofibroblastic differentiation markers including SMA and Vimentin are characteristically positive in PIMT but absent or only focally expressed in reactive inflammatory processes (2, 3). We acknowledge that the absence of these markers constitutes a diagnostic limitation. The biopsy was performed at an external institution whose standard immunohistochemical protocol did not include ALK, SMA, or Vimentin; our center was not involved in the pathological workup and could not supplement the panel retrospectively (Supplementary Figure 1). PIMT in its benign form carries a recognized risk of local recurrence, and ALK-negative subtypes may occasionally demonstrate metastatic behavior (3, 14, 15). The 10-month clinical course, during which the lesion regressed progressively without local invasion, distant spread, or recurrence, is compatible with a reactive inflammatory process (14, 15), but this clinical observation cannot substitute for histopathological marker-based differentiation between PIPT and PIMT. Definitive classification therefore could not be established, and the current diagnosis remains presumptive. In addition, the pathological slides and paraffin blocks are held by the external biopsy institution, and representative histopathological images (such as H&E staining and immunohistochemical staining) could not be obtained for this report. The original pathology report is provided as Supplementary Figure 1.

Spontaneous regression has been documented in PIPT, though such cases are uncommon (16). Most reported instances involved small lesions without high-risk imaging characteristics. In the present case, the lesion measured 3.4 cm × 2.6 cm and exhibited lobulation and vacuole sign, features rarely associated with spontaneous resolution. The lesion remained stable over the 2-month interval between April and June 2024, prior to TCM initiation, with no evidence of spontaneous reduction during this period. Following TCM intervention in July 2024, lesion size decreased sequentially: to 2.5 cm × 1.8 cm at 5 months and to 1.9 cm × 0.7 cm at 10 months. Lesion reduction was observed during each successive treatment phase, and cough and expectoration resolved in parallel. The pre-treatment stability of the lesion, its high-risk imaging profile, and the stepwise pattern of lesion reduction, each step aligning with a treatment phase, collectively favor a treatment-associated response over spontaneous resolution. However, this temporal association does not establish causation, and the contribution of spontaneous regression cannot be excluded.

The lesion also changed in shape over the follow-up period. At baseline it was ovoid (3.4 cm × 2.6 cm; long-to-short axis ratio approximately 1.3:1), whereas at the final review it was elongated (1.9 cm × 0.7 cm; ratio approximately 2.7:1). Both diameters decreased from baseline, confirming that the finding was not a redistribution of a stable mass. The greater reduction of the minor axis nonetheless indicates that fibrotic contraction and organization contributed to the change in morphology. Inflammatory pseudotumors contain variable amounts of fibrosis histologically, and during regression this component can contract to produce band-like or linear residual opacities (17). Spontaneous resolution of a pulmonary inflammatory pseudotumor has likewise been reported to leave a thin residual fibrotic band on follow-up CT (16). The residual elongated opacity in this case therefore likely represents organized fibrotic tissue accompanying true lesion reduction, rather than residual active inflammation. In TCM, PIPT is categorized within the disease entities of “cough” and “lung accumulation”, both characterized by a gradual progression from underlying Qi deficiency toward the accumulation of pathogenic excess (18, 19). The pathogenesis in this case advanced through three stages, each evidenced by discrete clinical findings. At onset, cough arising after upper respiratory infection indicated wind pathogen invading the lung with impairment of its dispersing and descending functions. With disease progression, lung Qi deficiency compromised spleen transportation and transformation, leading to phlegm-dampness accumulation. This was evidenced clinically by an enlarged tongue with teeth marks, white coating, and a deep pulse. In the later stage, sustained Qi deficiency and progressive phlegm obstruction impeded blood circulation, producing blood stasis as evidenced by sublingual vein distension. Qi stagnation, phlegm retention, and blood stasis mutually reinforced one another, ultimately leading to accumulation in the lung collaterals (20). Emotional constraint following the patient’s awareness of the pulmonary lesion further disrupted liver Qi dispersion, superimposing an additional layer of stagnation upon the pre-existing deficiency pattern (18).

Treatment was guided by the principle of supplementing healthy Qi while concurrently eliminating pathogenic factors, with the formula adjusted at each phase in accordance with the evolving syndrome pattern (Table 1). In Phase I, a modified Zhisou Powder, a classical formula for persistent cough following external pathogen invasion, was prescribed to disperse wind and restore lung diffusion. Huangqi was incorporated from the outset to consolidate Qi and pre-empt further deficiency, reflecting the classical strategy of simultaneously addressing the root and the branch in the early stage of pathogen invasion. In Phase II, as the clinical presentation shifted toward phlegm-blood stasis interconnection, wind-dispersing herbs were withdrawn, and herbs for resolving phlegm, dissipating masses, and promoting blood circulation were introduced. We increased the Huangqi dosage from 15 g to 24 g, following the classical principle of “cultivating earth to generate metal” (*pei tu sheng jin*, 培土生金), in which strengthening spleen Qi supports lung function and promotes clearance of accumulated pathological products (18). In Phase III, the core framework was maintained while Chaosuanzaoren was added to address insomnia, Yujin to relieve liver Qi constraint arising from emotional burden, and Fuchaoyiyiren (*Coicis Semen Praeparatum*) to reinforce spleen fortification. We maintained lung-spleen co-regulation throughout all three phases, together with phlegm resolution and blood activation. This approach aligns with strategies reported in the TCM literature on pulmonary accumulation disorders (19, 20).

The pharmacological rationale for the staged formula adjustments in this case maps onto the sequential pathogenic priorities identified at each treatment phase. In Phase I, when phlegm accumulation and dampness predominated, the formula centered on herbs with documented anti-inflammatory and immune-regulatory properties. In Phase II, as blood stasis emerged as the primary pattern alongside phlegm, herbs with demonstrated microcirculatory and anti-fibrotic actions were introduced. In Phase III, Qi-supplementing herbs were consolidated while anti-inflammatory support was maintained. The following preclinical data on selected individual components offer supporting biological plausibility for the formula-level rationale above, though they cannot substitute for direct evidence on the compound prescription itself. Huangqi was used across all three phases with stepwise dose escalation. In preclinical studies, it regulates T and NK cell activity, activates macrophages, and modulates cytokine expression, actions that may attenuate the chronic inflammation underlying PIPT (21). Danggui (*Angelicae Sinensis Radix*) and Chuanxiong (*Chuanxiong Rhizoma*), introduced in Phase II to address blood stasis, share ferulic acid as a principal bioactive constituent; combined extracts have been reported to exert antioxidant and anti-inflammatory effects, including downregulation of TNF-α, COX-2, and PGE2 in experimental inflammation models (22). In preclinical studies, Xiakucao suppresses TGF-β/Smad signaling and attenuates tissue fibrosis, effects relevant to the fibroblastic activity in PIPT (23). In Phase III, Yiyiren contributed immunomodulatory and anti-inflammatory effects through suppression of NF-κB, MAPK, and NLRP3 inflammasome pathways in macrophage models, complementing the spleen-fortifying rationale central to this treatment stage (24). These findings derive from preclinical studies of individual herbs or purified constituents. No pharmacological data on the specific compound formula used in this case are available. Differences in pharmacokinetics, multi-component interactions, and clinical dosing separate single-compound experiments from the therapeutic context of a multi-herb prescription. These data therefore provide biologically plausible hypothesis support rather than confirmatory mechanistic evidence for the clinical outcome observed.

We reviewed TCM case reports and clinical studies on PIPT over the past 25 years. Six publications comprising 16 patients were identified (Table 2) (25–30). Reported lesions were predominantly solitary nodules or masses of small-to-medium size, ranging from 2.0 cm × 1.5 cm (27) to 3.7 cm × 3.4 cm (29), with imaging features largely consistent with inflammatory or benign processes. Across these reports, a shared pathogenetic view emerged: PIPT was classified within the “lung accumulation” category and attributed to a root-deficiency-branch-excess pattern, in which lung and spleen Qi deficiency underlies the accumulation of phlegm, blood stasis, and heat toxin. Most cases showed lesion reduction or resolution after herbal intervention. The four individual case reports broadly followed syndrome differentiation and the principle of reinforcing healthy Qi while expelling pathogenic factors, though approaches varied considerably in the degree of individualization (25–27, 30). Wang prescribed a self-formulated Qingfei Jiedu decoction (清肺解毒方) for a pattern of phlegm stasis with heat toxin, centered on clearing heat and dissipating stasis without explicit Qi-supplementing or spleen-fortifying components (25). Qu used Huanglian Wendan Decoction (黄连温胆汤) as a fixed principal formula, with treatment organized around resolving phlegm and dissipating masses (29). Zhang et al administered Xuebijing injection (血必净注射液) as a standardized patent preparation, without syndrome-based individualization or dynamic adjustment to evolving clinical patterns (28). Follow-up durations were generally short, and TCM intervention in lesions of larger volume or higher imaging-based risk has seldom been reported. The existing evidence base is limited to six publications, most of which are individual case reports with variable reporting standards. Patient baseline characteristics, lesion features, outcome criteria, and follow-up durations differ considerably across studies, and these reports do not provide sufficient data for formal meta-analysis or systematic evaluation of confounding factors. The present case extends this literature in several respects. The initial lesion measured 3.4 cm × 2.6 cm and lacked unequivocal benign imaging features; surgical resection had already been recommended, placing diagnosis and treatment selection in a higher-risk clinical context than in most prior reports. Management followed a staged syndrome differentiation strategy that evolved with the underlying pathogenesis, progressing from wind-dispersing and lung-ventilating treatment in the early phase to Qi-supplementing and spleen-fortifying therapy with phlegm resolution and collateral activation in the later phases. Serial imaging over 10 months showed sustained lesion reduction, providing longer follow-up than previous TCM reports on this condition. Compared with prior reports, the present case is further distinguished by three features: a larger initial lesion with high-risk imaging characteristics in which percutaneous biopsy supported but could not definitively confirm PIPT, and surgical resection was declined; quantitative three-point CT tracking that enables a more rigorous assessment of lesion trajectory; and, most distinctively, a formally staged treatment protocol in which formula adjustments were guided by re-evaluated syndrome differentiation at each follow-up visit, with CT imaging serving as an objective measure of lesion change, rather than maintaining a fixed prescription or applying a standardized patent preparation. This structured approach may offer a more reproducible model for future prospective studies than those reported previously.

**TABLE 2:** Summary of case reports on TCM treatment for PIPT.

Author (Years)	Disease types	Lesion site	Lesion size (cm)	Primary pathogenesis	Core therapeutic principles	Core formula and herbs	Therapeutic outcome	Follow-up
Wang (25)	PIPT	Left upper lobe	2.0 × 1.5	Healthy Qi deficiency with pathogenic invasion, internal accumulation of phlegm-dampness, intermingling of phlegm and blood stasis.	Clearing the lung and detoxifying, resolving phlegm and removing blood stasis.	Self-designed Qingfei Jiedu decoction [Sangbaipi *(Cortex Mori*), Banxia, Fuling, Chenpi, Yuxingcao (*Herba Houttuyniae*), Baihuasheshecao (*Herba Hedyotis Diffusae*), Gualoupi (*Pericarpium Trichosanthis*), etc.]	Lesion undetectable on CT after approximately 7 weeks.	No recurrence after 2 months of continued treatment.
Zhang et al. (26)	PIMT	Trachea (subglottic to third tracheal ring, membranous wall)	Peanut-sized (not quantified)	Qi deficiency and blood stasis.	Supplementing Qi and fortifying the spleen, detoxifying and dissipating nodules.	Modified Huaji decoction [Huangqi, Shanyao (*Rhizoma Dioscoreae*), Chaobaizhu (*Rhizoma Atractylodis Macrocephalae*), Taizishen (*Radix Pseudostellariae*), Zaojiaoci, Xiakucao, Ezhu (*Rhizoma Curcumae*), etc.]	Dyspnea, cough, blood-streaked sputum, and night sweats resolved; laryngoscopy showed smooth tracheal mucosa with acceptable patency (5 months).	Not reported.
Wang et al. (27)	PIPT	Left upper lobe	3.7 × 3.4 × 3.0	Phlegm and stasis obstructing the lung, Qi deficiency of the lung and spleen.	Fortifying the spleen and resolving phlegm, clearing heat and detoxifying, removing stasis and dissipating nodules.	Self-designed YiQi Jianpi decoction [Taizishen, Chaobaizhu, Jiegeng, Qianhu (*Radix Peucedani*), Danshen (*Radix et Rhizoma Salviae Miltiorrhizae*), Banzhilian (*Herba Scutellariae Barbatae*), etc.]	Lesion nearly undetectable on CT after TCM treatment (3 weeks).	Recovery reported; no further imaging.
Zhang et al. (28)	PIPT	Left lung	Not mentioned	Heat toxin obstructing the lung, phlegm and stasis blocking collaterals.	Activating blood and removing stasis, softening hardness and dissipating nodules, dredging meridians and unblocking collaterals, ulcerating and dissipating heat toxin.	Xuebijing injection [Honghua (*Flos Carthami*), Chishao (*Radix Paeoniae Rubra*), Chuanxiong, Danggui, Danshen]	Cough and chest pain resolved; lesion nearly undetectable on CT after six 36-day treatment cycles.	CT normal at 6 and 20 months.
Qu et al. (29)	PIPT	Lung	Ranging from 1.0 to 5.5	Pain due to obstruction, intermingling of phlegm and blood stasis.	Unblocking orifices and removing phlegm-dampness, ventilating and descending lung Qi to unblock collaterals.	Modified Huanglian Wendan decoction [Huanglian (*Rhizoma Coptidis*), Zhuru (*Caulis Bambusae* in *Taenia*), Zhishi (*Fructus Aurantii Immaturus*), Banxia, Chenpi, Fuling, Ezhu, Sanleng (*Rhizoma Sparganii*), etc.]	Cough, chest pain, and hemoptysis resolved; shadows undetectable on CT/radiography (mean 29 days).	Not reported.
Zhu (30)	PIPT	Lung	Not specified	Qi deficiency of the lung and spleen, liver stagnation or *Yin* deficiency; intermingling of phlegm and blood stasis blocking the lung collaterals.	Supporting healthy Qi, resolving phlegm and removing stasis, softening hardness and dissipating accumulation.	Modified Liujunzi decoction / Xiaochaihu decoction / Yangyin Qingfei decoction; combined with Danggui, Taoren, Dannanxing (*Arisaema cum Bile*), Muli (*Concha Ostreae*), etc.	Cough, hemoptysis, and chest discomfort resolved; shadows undetectable after 4–5 months of treatment.	No residual lesion at 3 months.
Present case	Suspected PIPT	Right lower lobe	3.4 × 2.6	Healthy Qi deficiency as the root, intermingling of phlegm and blood stasis.	Staged syndrome differentiation, supplementing healthy Qi, expelling pathogens, activating blood, resolving phlegm and dissipating nodules.	Modified Zhisou Powder and Xiaoji Formula were sequentially: Jiegeng, Zaojiaoci, Xiakucao, Chenpi, Fabanxia, Huangqi, etc.	Longest diameter decreased from 3.4 to 1.9 cm (44% reduction); cross-sectional product decreased from 8.84 to 1.33 cm^2^ (85% reduction). Cough and expectoration resolved.	10 months; symptoms well controlled, lesion remained stable without progression.

TCM, Traditional Chinese Medicine; PIPT, pulmonary inflammatory pseudotumor; PIMT, pulmonary inflammatory myofibroblastic tumor.

## Limitations

As a single case report without randomized comparison, causal inference remains limited. The observed response is subject to variability in adherence, follow-up duration, and individual patient factors. Several diagnostic limitations warrant acknowledgment. The immunohistochemical panel performed at the external biopsy institution did not include ALK, SMA, or Vimentin, precluding definitive differentiation between PIPT and PIMT. Excisional biopsy was not performed because the patient declined surgical intervention, and serum angiotensin-converting enzyme levels were not measured; consequently, a localized sarcoid-like granulomatous process remains a diagnostic consideration. C-reactive protein (CRP) and erythrocyte sedimentation rate (ESR) were not monitored during the treatment period. Although these systemic markers have limited sensitivity for localized chronic inflammatory processes (31), even normal values may contribute to differential diagnostic evaluation by reducing the likelihood of active sarcoidosis or other systemic inflammatory conditions. In addition, the pathological slides and paraffin blocks are held by the external institution, and representative histopathological images could not be obtained for this report. The pharmacological rationale cited preclinical evidence from individual herbal components; no direct pharmacological data on the compound formula are available, and extrapolation from single-compound studies to multi-herb prescriptions remains a recognized limitation in TCM research.

## Conclusion

This patient presented with a 3.4 cm right lower lobe lesion showing lobulation and vacuole sign, without clear benign imaging features, which raised concern for malignancy. Percutaneous biopsy favored an inflammatory process consistent with PIPT; nevertheless, given the lesion size and the residual risk of malignant transformation, surgical resection remained the recommended approach for definitive diagnosis (8). The patient was fully informed of this recommendation and declined surgery, after which we initiated staged syndrome differentiation-based TCM management as conservative treatment. Serial CT imaging revealed stepwise lesion reduction over 10 months of follow-up. The longest diameter decreased from 3.4 cm at baseline to 1.9 cm (a 44% reduction), and the cross-sectional product of the two largest perpendicular diameters decreased from 8.84 cm^2^ to 1.33 cm^2^ (an 85% reduction). The lesion concurrently evolved from an ovoid to an elongated configuration, indicating that fibrotic contraction and organization accompanied this reduction; the residual elongated opacity likely represents organized fibrotic tissue rather than residual active inflammation. Cough and expectoration resolved in parallel. This case documents a temporal association between staged syndrome differentiation-based TCM management and progressive lesion reduction in an inflammatory pulmonary space-occupying lesion when surgery was declined. Whether this association reflects a treatment effect, spontaneous regression, or both cannot be determined from a single case. The observation may serve as a clinical reference point for future controlled investigation of conservative management strategies for PIPT.

## Data Availability

The raw data supporting the conclusions of this article will be made available by the authors, without undue reservation.
