# Supplementary material for: Staged traditional Chinese medicine treatment for suspected pulmonary inflammatory pseudotumor with progressive lesion reduction: a case report and literature review
Source: Front Med (Lausanne). 2026 Aug 20;13:1873013. doi: 10.3389/fmed.2026.1873013 (PMC13538104; doi:10.3389/fmed.2026.1873013)
Supplement: Supplementary file 1 [file Table_1.docx]

Supplementary Figure 1. Original pathology report from China-Japan Friendship Hospital (report date: June 24, 2024).

Notes: The immunohistochemical panel included TTF-1, NapsinA, P40, P53, CEA, Ki67 (MIB-1), KP-1, CD38, IgG, and IgG4; special stains included PAS, acid-fast, and silver staining. ALK, SMA, and Vimentin were not included in the panel. Patient-identifiable information has been redacted.
